# Prevalence of Prohibited Substance Use and Methods by Female Athletes: Evidence of Gender-Related Differences

**DOI:** 10.3389/fspor.2022.839976

**Published:** 2022-05-24

**Authors:** Katia Collomp, Magnus Ericsson, Nathan Bernier, Corinne Buisson

**Affiliations:** ^1^CIAMS, Université d'Orléans, Pôle STAPS, Orléans, France; ^2^CIAMS, Université Paris-Saclay, Faculté des Sciences du Sport, Orsay, France; ^3^Laboratoire AntiDopage Français, LADF, Université Paris-Saclay, Chatenay-Malabry, France

**Keywords:** doping, woman, anabolic agents, cannabinoids, beta-2 agonists

## Abstract

To achieve optimal sports performances, women and men may show specific doping practices because of the physiological and psychological gender differences, but there are few data on this topic. Here, we report the apparent use of prohibited substances and methods by female athletes based on analyses of the doping tests collected by the French Anti-Doping Agency from 2013 to 2019. We compared the frequency of use and the ergogenic and side effects to those of their male counterparts. The results revealed lower use of prohibited substances in female vs. male athletes, with significantly fewer anabolic agents, hormone and metabolic modulators, and cannabinoids. Gender specificity in utilization of substance classes was also shown. Relatively lower use of hormone modulators and cannabinoids and higher use of beta-2 agonists, diuretics and glucocorticoids were found in the woman cohort compared with men cohort, combined with the different choice of substances, possibly because of the altered ergogenic and/or side effects. However, no impact due to gender regarding the sports disciplines was observed, with both women and men showing similar use of anabolic agents, mainly in the anaerobic sports, and EPO and corticoids, mainly in endurance or mixed sports. Further studies are needed to put these French data into a global perspective, comparing uses across countries and exploring possible new developments in the fight against doping in women.

## Introduction

Women were first allowed to participate in the Olympic Games in the early 1900s, but only in a limited number of sports, such as archery, lawn tennis, figure skating, and swimming (Lal and Hoch, 2007). Since then, their inclusion in other sports has continued, but several reviews (Lepers, 2008; Thibault et al., 2010; Bassett et al., 2020) have noted that the significant gaps between women and men in terms of sports records persist, with an overall 10% average higher performance in men. This gap due to gender is essentially explained by physiological, psychological, and sexual characteristics, directly or indirectly linked to gonadal hormone secretion, i.e., estrogens and progesterone in women and testosterone in men (Bassett et al., 2020). Compared with men, women are smaller and lighter, and they have more body fat and lower skeletal muscle mass with shorter fiber lengths and cross-sectional areas (Haizlip et al., 2015; Trevino et al., 2019). In parallel, they have less bone mass and greater joint laxity (Nieves et al., 2005). All these factors mean not only lower force production and anaerobic capacity, but also a greater risk of bone fracture (Doherty et al., 2014; Wolf et al., 2015). Respiratory and cardiac functions are also lower in women during exercise because of a smaller respiratory tract and lungs coupled with a smaller heart and lower maximal cardiac output (Wheatley et al., 2014; LoMauro and Aliverti, 2018). The addition of lower blood volume, red blood cell number, and hemoglobin level leads to a significantly lower aerobic capacity compared with men, which is partially offset by a higher contribution of fat oxidation, lower fatigability, and higher recovery capability (Dasilva et al., 2011; Bassett et al., 2020). Last, sex steroid hormones influence cognitive function and emotional processing. According to the “sexually dimorphic” theory, men perform better in visuospatial skills, whereas women outperform men in verbal skills, and the impact of sex hormones at every level of the central nervous system to the muscle itself suggests a potential effect due to gender on balance and motor control (Castanier et al., 2021).

These sexual characteristics could lead to differences in doping behaviors and practices to achieve optimal performance. Since 2004, the World Anti-Doping Agency (WADA) (WADA prohibited list, 2022) has been updated at least once a year. It includes the classes of substances (S) and methods (M) prohibited at all times (in- and out-of-competition, only in-competition and in particular sports (P) (Supplementary Material section). To our knowledge, only one study (Mazzeo et al., 2019) conducted on Italian professional athletes, and focused on a direct comparison of substance use based on the results of a WADA-accredited anti-doping laboratory. In order to complete the results of this first descriptive study, we present here the larger scale findings on the detected use of banned substances and methods in samples from mostly elite athletes collected by the French Anti-Doping Agency (AFLD). The samples were analyzed by WADA-accredited laboratories in accordance with the WADA International Standard for Laboratories (WADA ISL, 2021). In parallel to the frequency of use, we reviewed the documented ergogenic and side effects for each substance class in women, in order to try to understand the differences in use between the genders, if any. We refer to female athletes based on their biological gender reported by the doping control officer.

## Methods

The data used for this study were based on test results obtained for doping controls (from the AFLD) performed between 2013 and 2019. The year 2020 was excluded from this study since fewer controls were performed, and many competitions were canceled due to COVID-19 pandemic. Data were extracted from the WADA Anti-Doping Administration & Management System (ADAMS) platform with AFLD consent. For each test result, the following information was collected: discipline, gender, test result [negative, adverse analytical findings (AAF, test results were prohibited substance or method have been confirmed) or atypical findings (ATF, test results where it is not possible to conclude as negative or AAF)], substance detected, substance or method class, sample type (urine or blood), and year of collection. As it was impossible to know the therapeutic use exemption (TUE) obtained by the athletes, all the AAF results reported by the laboratory, which followed the WADA International Standard for Laboratories (WADA ISL, 2021) in force along the selected years, were considered in this study, as in WADA's annual statistical report. This may be a study limitation, first, because some prohibited substances/methods are not currently detectable and, second, because there is a slight overestimation of the number of violations, particularly for beta-2 agonists and glucocorticoids that can be covered by a TUE. However, as shown in the article of Vernec and Healy (2020), this did not constitute a bias because the same prevalence of TUEs was observed for both genders, whatever the pharmacological class involved. In the same manner, due to the anonymization of the data, it was not possible to know if an athlete has been tested on different occasions but the same doping control logistics was followed by the AFLD regardless of the gender of the athletes. Finally, only one substance was reported when the drug and its metabolites were found in the same sample.

## Sports Classification

As there is a clearly sport-specific use of doping substances (Aguilar-Navarro et al., 2020), the sports were divided into the following categories: Category A: anaerobic sports (strength, power, and speed); Category B: aerobic sports (endurance); Category C: mixed aerobic/anaerobic sports; and Category D: combat sports and others. Team sports were included in Category C and sports with weight categories were included either in Category A (weightlifting, powerlifting, etc.) or Category D (boxing, judo, karate, etc.).

## Statistics

Standard descriptive statistics were performed in the R programming environment (version 4.1.1) and a Fisher exact test was used to compare the frequency distributions of: (i) use of substance classes relative to the number of samples per gender; (ii) use of substance classes relative to AAFs; and (iii) use of substance classes relative to sports categories. Due to the smaller number of samples taken from women vs. men, the statistical conditions were not fulfilled for some substances, for which it was therefore not possible to carry out the analysis of comparison. Similarly, S0 (non-approved substances), P1 (beta-blockers) and M2 (prohibited methods) were not processed due to the too small number of AAFs, i.e., 0 for S0, 5 for P1 (3 men; 2 women) and 1 for M2 (1 woman). The null hypothesis was rejected at *p* < 0.05.

## Results

### Number of Controls, AAFs, and Substances by Gender

The total number of urine and blood samples over the 7 investigated years (Table 1) was lower for women compared with men (22% of the total number of controls, *p* < 0.001). The number and percentage of AAFs related to the number of samples collected by the biological gender were also significantly lower in women vs. men when we compared the number of samples leading to AAFs (1.42 vs. 1.81%, *p* < 0.01) and the total number of substances identified in these samples (1.98 vs. 2.88%, *p* < 0.001). Compared to men (Table 2), significantly fewer anabolic agents (S1, *p* < 0.001) were reported from female samples (S1, *p* < 0.001), with lower values for both exogenous and pseudo-endogenous anabolic-androgenic steroids (AASs) (*p* < 0.001). In parallel, fewer hormone and metabolic modulators (S4, *p* < 0.001) and cannabinoids (S8, *p* < 0.001) were found in women vs. men, with no change in the other classes or methods. Last, there was a combined use of S1 (anabolic agents) and S4 (hormone and metabolic modulators) substances (71% of S4 substances associated with S1 substances) in men but not women.

**Table 1:** Number of controls (NC) for blood (B) and urine (U) and number of controls with AAF in males and females from 2013 to 2019.

		**Females**		**Males**	
		**NC**	**AAF**	**NC**	**AAF**
2013	B	78	0	634	1
	U	1817	30	5209	107
2014	B	98	1	342	1
	U	1537	24	5448	73
2015	B	101	0	451	0
	U	1578	26	5018	100
2016	B	136	0	554	9
	U	1572	18	5196	131
2017	B	93	0	764	2
	U	1085	31	4645	127
2018	B	38	0	438	4
	U	1487	18	5714	117
2019	B	89	1	346	1
	U	1527	11	5420	56
% of samples leading to AAF		**1.42%** ^++^		**1.81%**	

++ *p < 0.01, gender difference in % of samples leading to AAF*.

**Table 2:** Number and % per class for the most used substances (>3%).

**Class**	**Females: 223 (1.98%)** ^&&&^			**Males: 1157 (2.88%)**		
**Substances: Number and % per class for the most used substances (>3%)**						
S1	**70 (0.62%)** ^&&&^			**440 (1.09%)**		
	• 38 exogenous AAS^&&&^: stanozolol (39%), oxandrolone (15%), drostanolone (13%) • 14 pseudo endogenous AAS^&&&^^*^: 19-norandrosterone (47%), testosterone and related compounds^&^ (26.5%), boldenone^&^ (26.5%) • 18 other anabolics^**^ (26%): clenbuterol^*^(83%)			• 244 exogenous AAS: stanozolol (29%), trenbolone (13%), drostanolone and metanedienone (11%) • 142 pseudo endogenous AAS: 19-norandrosterone (46%), testosterone and related compounds (24%), boldenone (30%) • 54 other anabolics (12%): clenbuterol (94%)		
S2	**6 (0.05%)**			**41 (0.10%)**		
	• 6 rhEPO (100%)			• 29 rhEPO (71%) • 10 peptide hormones and releasing factors (24%) • 2 growth factors and growth factor modulators (5%)		
S3	14 (0.12%)^*^			**35 (0.09%)**		
	• 12 terbutaline^&^ (85.7%) • 2 others: salmeterol, vilanterol (14.3%)			• 19 terbutaline (54%) • 16 others: higenamine, salbutamol, salmeterol (46%)		
S4	**5 (0.04%)** ^&&&^^*^			**69 (0.17%)**		
	• 5 anti-estrogenic substances: tamoxifen (60%), raloxifene (20%), clomiphene (20%)			• 33 anti-estrogenic substances: tamoxifen (38%), raloxifene (9%) • 22 aromatase inhibitors: letrozole (9%), anastrozole (10%), androstatrienedione (11%),... • 14 metabolic modulators: meldonium (17%),…		
S5	32 (0.28%)^*^			**106 (0.26%)**		
	• 13 furosemide^&^^*^ (41%) • 10 canrenone (31%) • 2 hydrochlorothiazide (6%) • 7 others: other thiazides,…(22%)			• 23 furosemide (22%) • 20 canrenone (19%) • 23 hydrochlorothiazide (22%) • 40 others: other thiazides (8%), dorzolamide (6%),…		
S6	**29 (0.26%)**			**131 (0.33%)**		
	• 15 heptaminol^&&^^***^(52%) • 6 amfetamine and derivates (21%) • 6 tuaminoheptane (21%)		• 1 cocaine^&^^*^(3.0%) • 1 ephedrine and derivates (3.0%)	• 20 heptaminol (15%) • 45 amfetamine and derivates (34%) • 17 tuaminoheptane (13%)		• 27 cocaine (21%) • 16 ephedrine and derivates (12%) • 6 others (5%)
S7	**9 (0.08%)**			**34 (0.08%)**		
	• 8 morphine (89%) • 1 other: buprenorphine			• 29 morphine (85%) • 5 others: methadone (12%),...		
S8	**3 (0.04%)** ^&&&^^***^			**120 (0.30%)**		
	• 3 cannabis^&&&^(100%)			• 120 cannabis (100%)		
S9	52 (0.46%)^**^			**178 (0.44%)**		
	• 40 prednisone/prednisolone^&^^**^(77%) • 3 triamcinolone^&&^^**^ (6%) • 9 others: budesonide (11%), fluticasone (6%)			• 93 prednisone/prednisolone (52%) • 44 triamcinolone (25%) • 41 others: betamethasone (8%), budesonide (8%), …		
P1	**2 (0.02%)**			**3 (0.01%)**		
	• 2 propanolol			• 1 propanolol	• 1 bisoprolol	• 1 nebivolol
M2	**1 (0.01%)**			**0**		
	• 1 tampering					

&&& *p < 0.001;*

&& *p < 0.01;*

& *p < 0.05, gender difference in class and substance use relative to number of controls.*

*** *p < 0.001;*

** *p < 0.01;*

* *p < 0.05, gender difference in substance use relative to AAFs per class*.

### Gender Specificity in Percentage of Class Utilization

**Figure 1:**
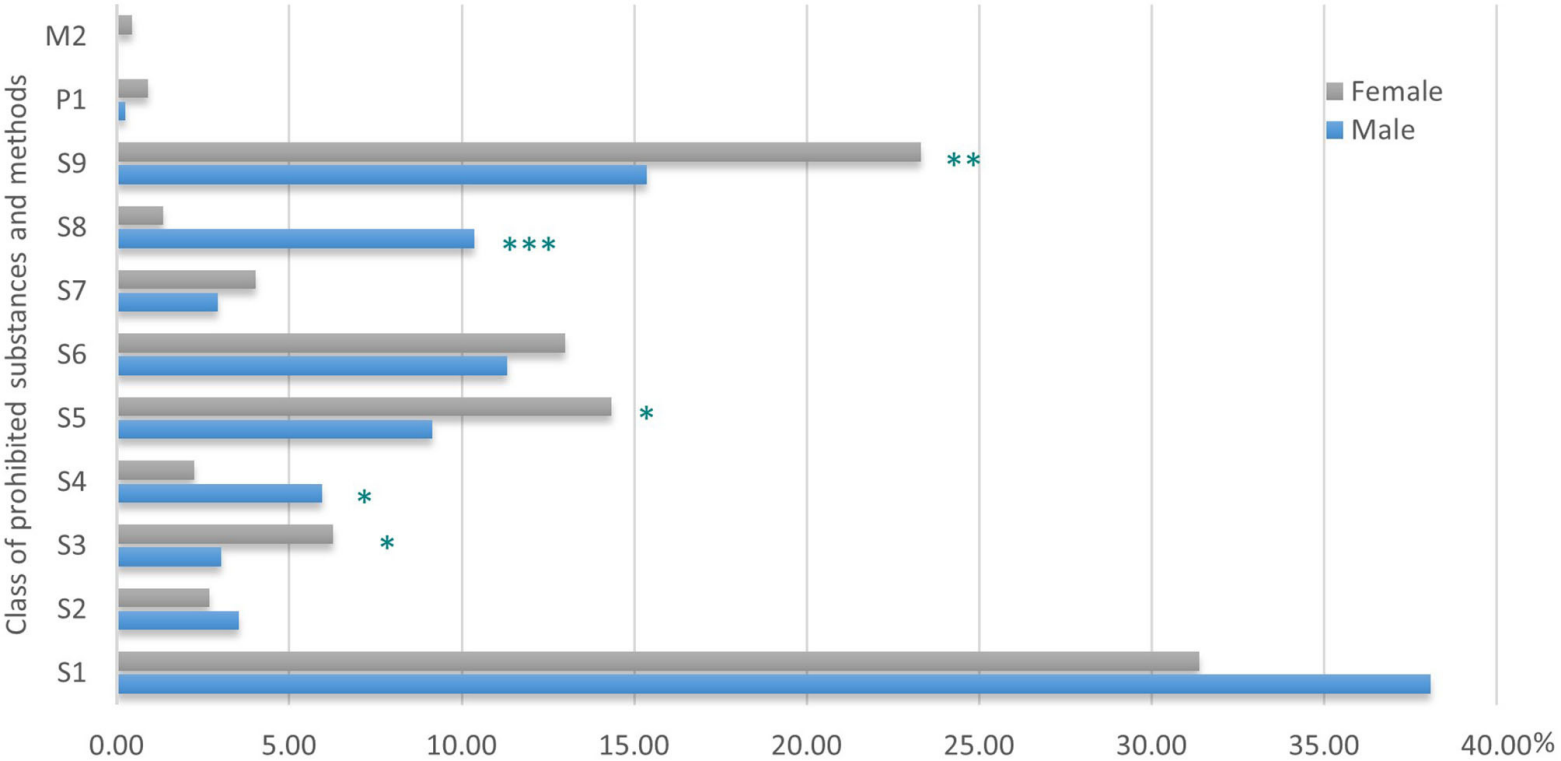
Distribution of classes of prohibited substances and methods (in %) in female and male athletes. ***p < 0.001; **p < 0.01; *p < 0.05, gender difference in distribution of class of substances and methods.

There was a significant difference due to gender in the relative percentage of use of the prohibited substance classes and methods (Figure 1). Indeed, compared to their male counterparts, female athletes used significantly fewer substances from S4 (*p* < 0.05) and S8 (*p* < 0.001) and more S3 (*p* < 0.05), S5 (*p* < 0.05), and S9 (*p* < 0.01), with a trend toward fewer S1 substances (*p* = 0.06). No difference due to gender in the % of use was found for the other classes.

### Gender Specificity in Substance Utilization

Significant differences due to gender were also found for some of the substances (Table 2). Among S1 substances, female athletes used relatively fewer endogenous AAS (*p* < 0.05) and more other anabolics (*p* < 0.01) like clenbuterol (*p* < 0.05) than men. In S3, the number of terbutaline uses relative to the number of controls was greater in women than in men (*p* < 0.05). In S5, furosemide was predominately detected in woman vs. man samples (*p* < 0.05). In S6, there was a preferential use of heptaminol in women (relative: *p* < 0.001; absolute: *p* < 0.01), with a significantly lower use of cocaine (*p* < 0.05) compared with men. In S9, the absolute and relative percentage of prednisone and prednisolone use was greater in women (*p* < 0.05 and *p* < 0.01, respectively), whereas the use of triamcinolone was significantly lower (*p* < 0.01). In S2, women exclusively used recombinant EPO, in contrast to men, in whom hGH, LH-releasing factors, and growth factors were detected. Similarly, in S4, only anti-estrogenic substances such as tamoxifen were found in women, although they were only used by half the men, who combined them with aromatase inhibitors and metabolic modulators such as meldonium.

### Gender Specificity in Sports Categories

**Figure 2:**
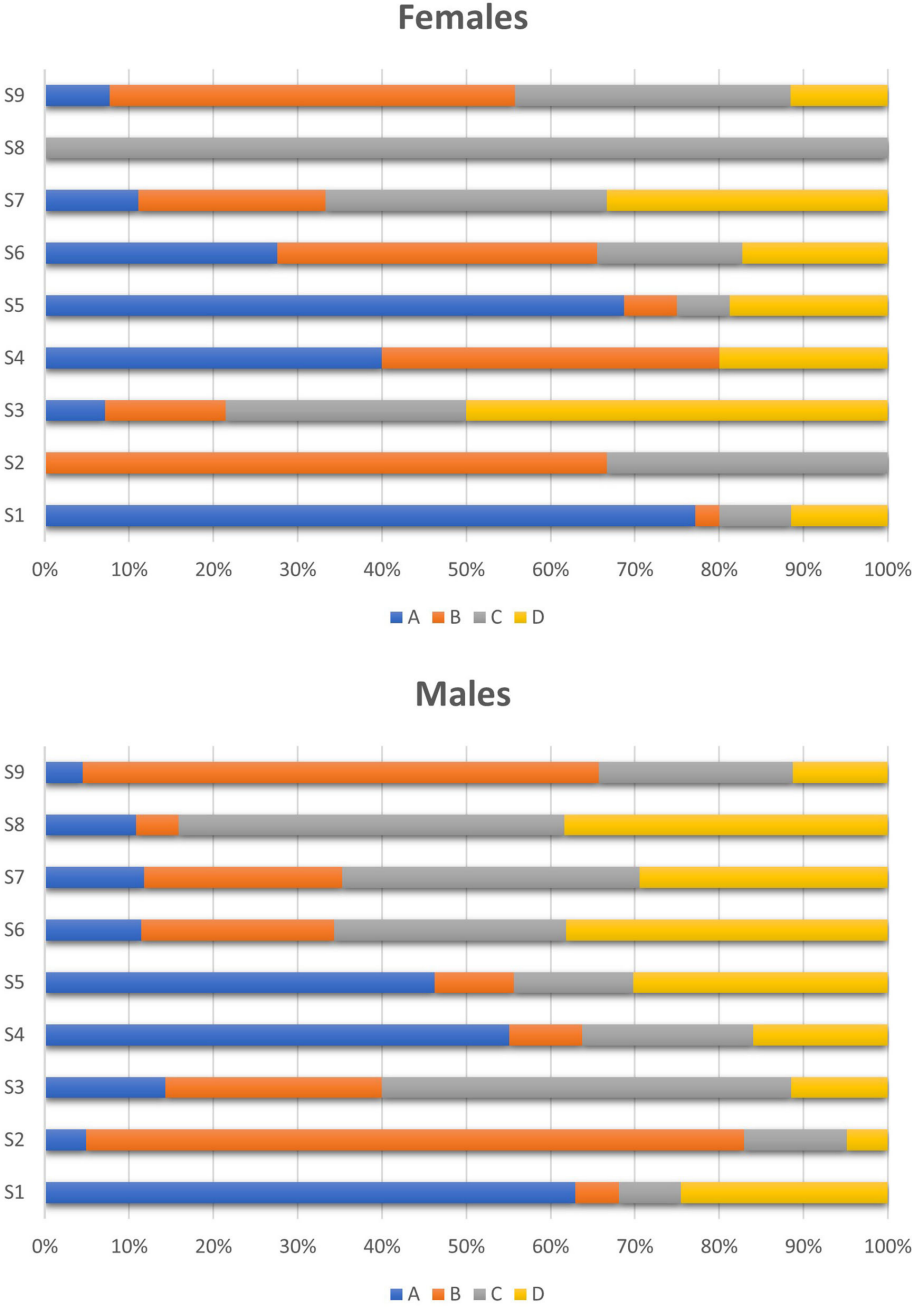
Class use by women and men regarding the sports discipline. Group A: anaerobic sports (strength, power, and speed); Group B: aerobic sports (endurance); Group C: mixed aerobic/anaerobic sports; Group D: combat sports and others. S0, P1 and M2 were not represented, due to the too small number of AAFs, i.e., 0 for S0, 5 for P1 (3 men, 2 women) and 1 for M2 (1 woman).

No gender difference emerged in class use regarding the sports categories (Figure 2). Indeed, in both female and male athletes, S1 class was mainly used in anaerobic sports and S2 class in aerobic sports. S5 class was mostly found in weight-category sports in both women (Category A: 53%; Category D: 14%) and men (Category A: 40%; Category D: 19%), whereas S9 class was mainly reported in endurance and mixed sports.

## Discussion

The main results of this study of female vs. male athletes were the following: (i) lower use of prohibited substances/methods for females, with fewer anabolic agents, hormone and metabolic modulators, and cannabinoids; (ii) specific use of substance classes and prohibited methods, including a relatively lower use of cannabinoids and hormone modulators for females, and also higher use of beta-2 agonists, diuretics, and glucocorticoids; (iii) a different choice of substances, possibly due to altered ergogenic and/or side effects; and (iv) an identical class use according to the sports categories, with anabolic agents and diuretics mainly found in anaerobic sports, and peptide hormones and glucocorticoids in endurance or mixed sports.

Our data showed a lower number of tests (22%) in female than male athletes, which partly reflects the lower number of elite French female athletes (39%) (French Ministry of Sports, 2021), and a lower percentage of AAFs, because of fewer anabolic agents (S1), hormone and metabolic modulators (S4), and cannabinoids (S8). Our data were in accordance with the descriptive study of Mazzeo et al. (2019) on Italian professional athletes from 2007 to 2017, but comparison is difficult because of the smaller cohort in that study. Given the gender differences in class use noted here, we now present the known ergogenic and side effects for each class in women, analyzing, where possible, substance use and effect within those classes.

### S1. Anabolic Agents

Our data showed a significant difference in the utilization of S1 substances, with lower use by women vs. men, because of the significantly lower number of both exogenous and pseudo-endogenous AAS adverse cases. Short-term administration of anabolic substances improves male anaerobic performance through an increase in muscle strength and power (Hartgens and Kuipers, 2004), but it does not improve aerobic performance (Baume et al., 2006). The few studies performed in women seem to show, as in men, a significant increase in muscle mass combined with a significant improvement in anaerobic performance (Franke and Berendonk, 1997; Fitch, 2008; Huang and Basaria, 2018), which may explain the predominant use of S1 class in anaerobic sports for both women and men. It was interesting to note, however, the relatively greater use by females of “other anabolic agents.” AAS induce abnormal endogenous hormone secretion, producing such reversible or irreversible damage as acne vulgaris, androgenic alopecia, hypertrichosis, liver cancer, cardiovascular risks, renal failure, and increased tendon ruptures (Hartgens and Kuipers, 2004). AAS are also known to induce psycho-behavioral disorders leading to violence or depression, with more aggressive responses in men (Gruber and Pope, 2000; Chegeni et al., 2021). AAS also induce gender-specific side effects, with either gynecomastia, testicular atrophy, azoospermia, and infertility in men or amenorrhea, uterine atrophy, and clitoral enlargement in women (Liu and Wu, 2019). There is a consensus that the use of classic AAS such as testosterone and nandrolone esters is associated with greater physiological adverse effects in females than male athletes, with hirsutism, voice deepening, and menstrual disturbances, though this depends on the molecule (Franke and Berendonk, 1997; Fitch, 2008; Huang and Basaria, 2018). Use of other AAS considered to be steroid precursors, such as DHEA, has been less documented in a young healthy population but seems to induce fewer ergogenic, physiological and psychological side effects in recreationally trained female athletes (Gravisse et al., 2018). However, the physiological side effects of other non-androgenic anabolic agents such as clenbuterol appear to be more limited than those of AAS in female athletes. Despite the lack of studies in woman, the absence of androgenic effects of this type of substance could explain its greater use by female athletes demonstrated here.

### S2. Peptide Hormones, Growth Factors, Related Substances, and Mimetics

No significant difference in the use of the S2 class between women and men was observed in our data. Short-term use of rhEPO markedly improves endurance capacity in male athletes, and this is identified by a significant increase in VO_2_ max or maximal aerobic power in running or cycling trial performances (Salamin et al., 2018; Sgrò et al., 2018; Haile et al., 2019). For this reason, rhEPO analyses focus on aerobic sports and, unsurprisingly, we found all rhEPO cases in either endurance or mixed sports. It is generally assumed that the main part of performance improvement with rhEPO administration is related to blood adaptations that enable higher oxygen transport. Unfortunately, to our knowledge, no study on performances in female athletes has been conducted, even though their lower blood volume, red blood cell number, and hemoglobin level probably modulate the stimulating effect on erythropoiesis. Regarding the side effects, they appear to be much more limited in the short term since the advent of micro-dose administration, but here again, data exist only for male athletes (Salamin et al., 2018). In a study on young healthy sedentary volunteers, however, Gambardella et al. (2016) suggested that the rhEPO effects might be confined to the vascular wall in males, with stabilization of the thrombi, whereas its effects in females can be observed in the peripheral circulation, with possible high blood thrombogenicity that merits more careful medical attention to female athletes. Nevertheless, rhEPO accounted for all the AAF in females for this S2 class, contrarily to males. Caution is warranted here in view of the small number of analyses carried out, but it could be suggested that the other S2 substances used by male athletes, i.e., hypoxia-inducible factors, GH, LH and their releasing factors, may induce either less ergogenic or more marked side effects in women. Given the difficulty of obtaining approval from the Ethics Committees for the administration of rhGH in athletes, few studies have investigated its ergogenic effects in trained subjects (Berggren et al., 2005; Marchand et al., 2019), with only one in females (Berggren et al., 2005). The authors reported no change in maximal oxygen uptake or maximum power output during exercise with rhGH use (Berggren et al., 2005; Marchand et al., 2019), and also increased body weight that was attributed to fluid retention and not muscle mass (Berggren et al., 2005). Despite these results, the rhGH impact on both anaerobic and aerobic performance is not in doubt, due to its direct and indirect anabolic (Holt and Sönksen, 2008; Siebert and Rao, 2018) and lipolytic effects (Healy et al., 2006) with, in terms of IGF-1, a greater response to rhGH treatment in men (Giannoulis et al., 2005). Although it remains unknown whether the cardiovascular, metabolic, and neuropsychiatric side effects reported in patients with chronic GH treatment occur in athletes (Siebert and Rao, 2018), short-term supra-physiological rhGH treatment was shown to induce central hypothyroidism in both male and female recreationally trained athletes from various team sports, but with distinct gender-related patterns, probably due to modulation of gonadal steroids on the GH-IGF-1 axis (Sgrò et al., 2016). Last, it should be recalled that, given the small number of GH analyses and the very short elimination half-time of rhGH (Giannoulis et al., 2005), the number of positive cases probably greatly underestimates actual use by athletes, irrespective of gender.

### S3. Beta-2 Agonists

This study highlighted relatively greater use of beta-2 agonists in women vs. men, with a higher use of terbutaline. The effects of beta-2 agonists on aerobic performance have been investigated in both male and female athletes after acute therapeutic inhalation of salbutamol (Koch et al., 2015, 2016) and terbutaline (Molphy et al., 2019), with no performance change noted whatever the gender (Koch et al., 2015, 2016; Molphy et al., 2019). However, acute salbutamol intake induced a significant increase in both anaerobic peak power and capacity in women (Le Panse et al., 2007), whereas only improvement in peak power was reported in men (Collomp et al., 2005). After short-term oral salbutamol administration, maximal anaerobic power but not capacity was improved, irrespective of the gender or training status, with no change in body composition (Le Panse et al., 2005, 2006). Other studies performed only in men showed various results after salbutamol or terbutaline inhalation or intake, with change or no change in aerobic (Collomp et al., 2000) and anaerobic performances, coupled or not to anabolic effects (Hostrup et al., 2014; Jessen et al., 2021). Women may have greater sensitivity to beta-2 receptor stimulation (Kneale et al., 2000), but in view of the same TUE prevalence for beta-2 agonists in female and male athletes (Vernec and Healy, 2020) and the lack of studies on the pharmacokinetics and the ergogenic, physiological, and psychological terbutaline effects in women, it is difficult to speculate on the higher number of terbutaline cases found in female athletes. Last, in the abovementioned studies, some participants of both genders experienced mild and similar adverse physiological side effects, whereas psychological repercussions were not explored.

### S4. Hormone and Metabolic Modulators

Lower use of this class by women was clearly shown in this study. Over the 7 years investigated, the most frequently used substance by both genders was tamoxifen, which is an estrogen antagonist prescribed for the breast cancer in women that stimulates testosterone secretion in men but not women. Tamoxifen's effect on athletic performance remains unknown (Matich, 2007), but its estrogen effect seems to be mostly used by men to limit the gynecomastia induced by AAS administration, as most samples of men with S4 substances also contained S1 substances.

### S5. Diuretics and Masking Agents

Our data showed a relatively greater use of the S5 class by the women, with a higher number of furosemide cases in this population. S5 substances have no ergogenic effect and are used to mask the use of other prohibited substances by diluting the urine, or methods, to induce rapid weight loss or treat hypertension. The majority of these cases was thus found, regardless of gender, in the anaerobic and combat sports that have weight categories. It can be suggested that the relatively higher use by women may be related to the greater variations in body weight because of the fluctuations in hormonal status during the menstrual cycle (Ryan et al., 2021).

### S6. Stimulants

The data did not show gender-specific effects for the S6 class but did show gender specificity for the substances, with less cocaine and more heptaminol in women vs. men. Several studies have shown that amphetamine and cocaine administration improves aerobic and anaerobic performances in humans (Wyndham et al., 1971; Chandler and Blair, 1980; Clarkson and Thompson, 1997), with a tolerance and withdrawal effect when taken chronically, but no studies have been conducted in woman. For ephedrines, a correlation clearly exists between the dose administered and its ergogenic effects (Trinh et al., 2015). Some studies have been performed in females (Clemons and Crosby, 1993; Chait, 1994), with one showing a better mood response to ephedrines in male subjects (Chait, 1994). In this study, the lower number and % of female samples containing cocaine metabolites compared with male samples suggest that females use fewer illicit drugs than male. Regarding the higher number of samples containing heptaminol in women and the lack of studies on the ergogenic and side effects of this substance in either gender, it can only be hypothesized that women tend to use more supplements, with many of them containing heptaminol.

### S7. Narcotics

As for the S6 class, the data showed no impact of gender on the absolute or relative use of substances from the S7 class. Morphine, a major analgesic and metabolic of codeine, accounted for most of the cases of this class in both women and men.

### S8. Cannabinoids

It appears clearly from our data that the use of cannabis is more limited in women. These data are in line with the various studies carried out by questionnaire (Lorente et al., 2005), showing a lower use of “recreational drugs” for women compared with men, even outside the sporting context. As there is no consensus at present about the ergogenic effect of cannabis, the lower use in women probably reflects a healthier lifestyle. Last, it should be noted that most of the cannabis cases in male athletes were found in mixed sports, including team sports and combat sports.

### S9. Glucocorticoids (GCs)

The data showed a relatively higher use of glucocorticoids by women vs. men, with a greater number of prednisone and prednisolone samples and a lower number of triamcinolone samples. Only systemic and not local (Kuipers et al., 2008) short-term administration of corticoids produces significant ergogenic effects during exercise lasting more than 40 min in both man (Arlettaz et al., 2007; Collomp et al., 2008) and woman (Le Panse et al., 2009) recreationally trained athletes, with a similar gender performance improvement, whereas GC ergogenic effects appear more variable in brief exercise (Nordsborg et al., 2008; Casuso et al., 2014; Zorgati et al., 2014). This was clearly evident in our data by the predominance of GCs in endurance and mixed sports, regardless of gender. The duration of hypothalamic-pituitary-adrenal (HPA) axis inhibition with oral therapeutic doses of prednisone/prednisolone was also found to be similar in women and men (Jollin et al., 2010; Collomp et al., 2014). However, as no hyperglycemia has been reported in female athletes after short-term prednisone treatment (Le Panse et al., 2009), contrarily to their male counterparts (Arlettaz et al., 2007), it could be suggested that women are less sensitive than men to the insulin resistance induced by GCs. This may be an argument for the preferential use of GCs in women, as similar TUE prevalence for GC was reported in elite athletes of both genders (Vernec and Healy, 2020).

## Limitations

First, the prevalence of drug use was estimated from the detection in anti-doping tests. This is an extrapolation, but the preferred approach for estimating the prevalence of illicit drug use in different populations, as the results from toxicological analyses of biological matrices are much more conclusive than the use of questionnaires, which leads to underestimation (Tavella et al., 2020).

Second, it was assumed that the detection of substances was identical in women and men. Most drugs currently in use were approved based on the clinical trials conducted only on men, despite the fact that pharmacokinetics may be gender specific, as it was recently shown for anti-inflammatory drugs, antidepressants, and anti-cancer drugs (Zucker and Prendergast, 2020) with, in general in women, higher blood concentrations inducing higher risk of adverse effects, reduced clearance and longer elimination times, that may potentially increase AAF female cases in an anti-doping context.

Third, it cannot be ruled out that women may be using drugs with shorter half-lives, and hence less frequent detection, than men, or *vice-versa*, for all the prohibited classes.

At last, considering the fewer samples collected from women than from men, a conservative test for unequal sample sizes was selected, but it was unfortunately not possible to statistically compare the gender-related use for all the detected substances.

Therefore, in order to definitively and more sharply establish the gender-specific doping prevalence, it seems necessary to set up a new anti-doping approach better targeted on female athletes, according to their hormonal status if possible.

## Conclusion and Perspectives

In conclusion, despite the smaller number of controls carried out for women compared with men, the present data highlight the apparent lower use of prohibited substances by women vs. men athletes. We also found gender-related differences in the use of substances and classes of substances. However, no gender difference related to the type of sport was observed, with anabolic agents mainly found in the anaerobic sports, and rhEPO and corticoids in endurance or mixed sports. Further studies are required to collaborate these French data into a global perspective, comparing uses across countries and opening discussions on possible new developments in the fight against women doping. Areas for discussion include increasing the number of tests in this population and the need for further study of the pharmacokinetics and the physiological, psychological, and side effects of many of the substances that remain unknown in woman athletes.

## Data Availability Statement

The original contributions presented in the study are included in the article/Supplementary Material, further inquiries can be directed to the corresponding authors.

## Author Contributions

KC and CB: study conception, data analyses, and manuscript writing. ME and NB: data analyses and manuscript writing. All authors agreed to the published version of the manuscript and formed a consensus on the resulting conclusions and recommendations.

## Conflict of Interest

The authors declare that the research was conducted in the absence of any commercial or financial relationships that could be construed as a potential conflict of interest.

## Publisher's Note

All claims expressed in this article are solely those of the authors and do not necessarily represent those of their affiliated organizations, or those of the publisher, the editors and the reviewers. Any product that may be evaluated in this article, or claim that may be made by its manufacturer, is not guaranteed or endorsed by the publisher.
