# Supplementary material for: Prevalence of Prohibited Substance Use and Methods by Female Athletes: Evidence of Gender-Related Differences
Source: Front Sports Act Living. 2022 May 24;4:839976. doi: 10.3389/fspor.2022.839976 (PMC9172204; doi:10.3389/fspor.2022.839976)
Supplement: Supplementary file 1 [file Table_1.docx]

Supplementary material part

**WADA classes of prohibited substances and methods**

| S0 | Non-approved substances |
| --- | --- |
| S1 | Anabolic agents |
| S2 | Peptide hormones, growth factors, related substances, and mimetics |
| S3 | Beta-2 agonists |
| S4 | Hormone and metabolic modulators |
| S5 | Diuretics and masking agents |
| S6 | Stimulants |
| S7 | Narcotics |
| S8 | Cannabinoids |
| S9 | Glucocorticoids |
| P1 | Beta-blockers |
| M1-M2-M3 | Prohibited Methods |
